# The “Butter Tree” ( *Diploknema butyracea* ) for Sustainable Food Systems and Functional Applications

**DOI:** 10.1002/pei3.70129

**Published:** 2026-02-15

**Authors:** Prekshya Timsina, Diksha Timsina, Navin Gautam, Ashma Subedi, Kishor Rai, Sangam Dahal

**Affiliations:** ^1^ Department of Food Technology, Central Campus of Technology Tribhuvan University Dharan Nepal; ^2^ Food Safety & Quality Assurance Management, Lambton College Toronto Ontario Canada; ^3^ London Geller College of Hospitality and Tourism University of West London London UK; ^4^ Central Department of Food Technology Tribhuvan University Dharan Nepal; ^5^ Department of Food Technology and Quality Control Ministry of Agriculture and Livestock Development, Food Technology and Quality Control Division Office Biratnagar Nepal

**Keywords:** butter tree, chiuri, industrial applications, pharmacological, phytochemicals, sustainable food

## Abstract

*Diploknema butyracea* , also known as the Himalayan butter tree, is mainly valued for its butter‐producing seeds and ecological significance. In addition to being significant for its traditional usage, it has lately gained popularity in the food, cosmetics, and pharmaceutical industries for its pharmacological and therapeutic significance. All components of the tree contain beneficial phytochemicals, including phenolics, flavonoids, ascorbic acid, and essential fatty acids, and possess bioactive properties, including anti‐inflammatory, antifungal, antioxidant, and antibacterial properties. Chiuri seeds contain over 60% fat and are used to produce chiuri butter with skin‐healing properties and industrial applications. Despite being culturally integrated with its economic and medicinal importance, *D. butyracea* faces challenges from deforestation and overexploitation. This plant is underexplored and not widely promoted in accordance with its worth. This review describes the physicochemical and phytochemical composition of different components of *D. butyracea* . Detailed study of literature for this review was performed using databases like PubMed, Web of Science (WOS), and Scopus. Moreover, the botanical description, ecology and distribution, processing methods, and medicinal and industrial applications are also discussed, providing insight into its potential for sustainable development in the region. This review aims to emphasize the multifaceted importance of *D. butyracea* from all the available information and encourage further exploration into its commercial importance in sustainable food systems and ecological potential.

## Graphical abstract

The graphical abstract figure highlights *Diploknema butyracea* , its production as well as its industrial application, traditional use, and pharmacological properties.

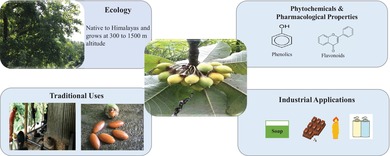

## 1 Introduction

*D. butyracea* Roxb often called as “butter nut tree” (Chiuri), is a medium‐sized deciduous tree with a straight trunk and a member of Sapotaceae family. It stands around 20 m tall (Tiwari et al. 2020). This plant, indigenous to the Himalayan area, is found in Nepal, India, and Bhutan at elevations between 300 and 1500 m. In Nepal, it is most commonly known as “Chiuri”; in English, as “Indian butter nut”; and in India, as “Chiuri” or “Phulwara” (Tyagi 2015). The tree is frequently found near human settlements in wastelands, pastures, and farmed fields. *D. butyracea* naturally inhabits temperatures between 24°C and 27°C (Zargar and Kumar 2018).

Economically significant genera *Diploknema*, *Manilkara*, *Pouteria*, *Chrysophyllum*, *Madhuca*, and others are part of this latex‐producing family, which is dispersed throughout the tropical world according to habitat (Press et al. 2000). Taxonomically, *D. butyracea* Roxb. belongs to the kingdom Plantae, sub‐kingdom Angiospermae, order Ericales, family Sapotaceae, sub‐family Sapotoideae, and genus *Diploknema* (Thapa 2019). It is indigenous to southwestern China, the Himalayas, and Southeast Asia. Of these seven species, only *D. butyracea* is found in Nepal (Shrestha et al. 2022). Different ethnic groups in Nepal use different parts of the chiuri plant for herbal remedies and other uses (Tiwari et al. 2020). Chepang, a native ethnic group (Tibeto‐Burman) in Nepal, utilizes *D. butyracea* for medical purposes. With an average of 67.94 kg per tree, fruit production ranges from 5 to 694 kg (Joshi 2010). Fruits and seeds are nutrient‐dense and contain high concentrations of phytochemicals with antioxidant qualities (Bvenura and Sivakumar 2017). The most valuable aspect of this plant is its seeds, which are commonly referred to as “Chiuri ghee” due to their almost 65% oil/fat content (Tamta and Tewari 2018). Oil yield ranges from 42% to 47% of seed weight. It is white, ghee‐like in consistency, and has a pleasant flavor and smell (Dhakal 2014). Methyl ester of palmitic acid, oleic acid, linoleic acid, and stearic acid are the major components found in seed fat (Devkota et al. 2012). Seed fat can also be used as an emollient for wintertime chapped hands and feet and as an ointment for rheumatism, boils, pimples, and burns. The bark of the tree is used to cure leprosy, diabetes, rheumatic pain, tonsil inflammation, asthma, indigestion, ulcers, itching, bleeding, limb contraction, and wounds (Rijal 2011). The parts—bark, leaves, flowers, seeds, and fruits—contain a wide range of phytoconstituents, such as tannins, alkaloids, flavonoids, steroids, terpenoids, and palmitic acid, in addition to vital nutrients like calcium, potassium, sodium, magnesium, zinc, and different sugars that exhibit a variety of pharmacological and medicinal properties. Beyond its conventional use, *Diploknema* has a wide range of industrial uses in cosmetics, medicines, confections, and nutraceuticals (Anand et al. 2024).

An estimated 20 tons of chiuri butter was exported from Nepal to the European market per year between 2011 and 2016 (Bhattarai et al. 2021). Fruit's nutritional potential and medical significance, especially its bioactive properties, have led to the usage of fruit in functional foods, according to earlier research (Dawadi et al. 2023). *D. butyracea* is in danger of going extinct due to constant human pressure, despite its enormous economic and therapeutic benefit (Biswas et al. 2003). These plant species produce a sufficient number of seeds; however, regeneration remains inadequate (Khanka et al. 2009). Even though the species is traditionally grown in and around villages due to its enormous economic value, systematic plantation of its high‐yielding genotypes on community forests or lands will produce enough edible (fruits and seeds) and fodder (leaves) components, thereby enhancing the nutritional and livelihood security of the people living in remote and underdeveloped areas of the state (Joshi, Joshi, et al. 2018). However, due to pressures brought on by humans, *D. butyracea* is in danger of extinction despite its significant economic and medical value (Joshi, Chaudhary, and Rawat 2018).

Despite its wide range of traditional uses and ecological benefits, *D. butyracea* remains significantly underutilized and under‐researched in the scientific and industrial domains. There is a notable lack of comprehensive data on its phytochemical constituents, nutritional profile, and mechanisms behind its reported bioactivities. Modern food science has yet to fully explore the functional properties of chiuri butter as a sustainable alternative. Additionally, limited studies have examined optimized processing techniques for oil extraction, deodorization, and product development suitable for consumer markets. The role of *D. butyracea* in agroforestry, climate resilience, and rural livelihoods has also received minimal attention in sustainability‐focused research. Furthermore, a lack of awareness, standardization, and market integration hinders its potential adoption in mainstream food, cosmetic, and nutraceutical industries. These gaps highlight the need for multidisciplinary research efforts to valorize *D. butyracea* within the frameworks of sustainable food systems, functional ingredient innovation, and circular bioeconomy models. This review paper summarizes findings of functional multidisciplinary studies from perspectives which could present the gap and shape the studies to be conducted in the near future.

## 2 Description of *D. butyracea* (Chiuri)

*D. butyracea* is a naturally occurring part of broadleaved forests and can be found alone or in small groups, especially in association with buddhairo (*Lagerstroemia parviflora*), dhayo (*Woodfordia fruticosa*), sal ( *Shorea robusta* ), tanki ( *Bauhinia purpurea* ), barro ( *Terminalia bellirica* ), amla (*Embelica officinalis*), tatari (*Dillenia pentagyna*), and Bhalayo ( *Rhus succedanea* ). The three main related species are mugwort (*Artemisia indica*), currant (*Ribes takare*), and artillery plant (*Pilea symmena*) (Bhattarai et al. 2021). It is a multipurpose tree species that is commercially significant, nutritionally significant, slow‐growing in the environment, and culturally integrated (Bhattarai et al. 2024). The flowers are fragrant and have a yellow hue. The fruit has three seeds and is round in form. October–November is when chiuri flowers begin to bloom, and June–July is when the fruits begin to ripen. The seed has a significant fat content called Phulwara butter. The average yearly rainfall in its natural environment is between 1000 and 2000 mm (Zargar and Kumar 2018).

### 2.1 Botanical Morphological Description of *D. butyracea*

-
*D. butyracea* tree is about 25 m tall.
- Branchlets are robust, striate, yellowish brown, terete to subterete, or brown pubescent to subglabrous with lenticels.
- The lanceolate stipules are brown to pale yellow, early deciduous, and measure about 5 mm.
- Leaf blades are elliptic‐oblong to ovate or ovate‐oblong in shape, (6–)17–35 cm (3–)8–17 cm; leathery in texture, with yellowish‐brown to brown pubescence, a cuneate base and an obtuse to obtuse‐acuminate apex.
- Flowers are axillary, single to six in number.
- Pedicel is about 2–4.5 cm, pubescent, 5 cm in fruit.
- Sepals are oval (4 or 5) or 6, 0.9–1.5 × 0.6–1 cm, hairy on the exterior, scattered lanate on the interior, and obtuse at the apex.
- The corolla is 1.5–2 cm in length and has 8–10 oblong, ovate, or slightly obovate lobes; the apex is obtuse to acute, and the border is often irregularly crenulate.
- Stamens are glabrous or brown lanate, with sagittate, awned anthers.
- The ovary is conical, varying in size from 2 to 5 mm, rust‐colored and sericeous.
- Style is glabrous (1.5–5 cm).
- Fruit is ovoid‐globose to oblong, 2–2.5 × 1–1.5 cm, smooth, apex acute, exocarp fleshy, and 1–3(–5)‐seeded.
- Seeds are brown, oblong–obovoid, about 1.3 × 1 × 0.6 cm, smooth and lustrous.

*Source*: Bhattarai et al. (2021).

The physical characteristics of *D. butyracea* fruit as reported in previous studies are shown in Table 1.

**TABLE 1:** Physical characteristics of *D. butyracea* fruit.

Attributes	Reported values	References
Color	Green to yellow when ripe	Bhattarai et al. (2024) and Sherpa et al. (2017)
Shape	Oval to ellipsoid	Bhattarai et al. (2024) and Sherpa et al. (2017)
Weight (g)	5.02–11.24	Bhattarai et al. (2024) and Bhutia et al. (2018)
Length (cm)	3.24	Bhattarai et al. (2024)
Width (cm)	2.8	Bhattarai et al. (2024)
Diameter (cm)	2.5–3.7	Bhattarai et al. (2024)
Specific gravity	0.8–1.2	Bhutia et al. (2018)
Peel weight	0.07–2.07	Bhutia et al. (2018)
Pulp to seed ratio	1.47–2.38	Bhutia et al. (2018)

### 2.2 Ecology and Distribution of Chiuri

**FIGURE 1:**
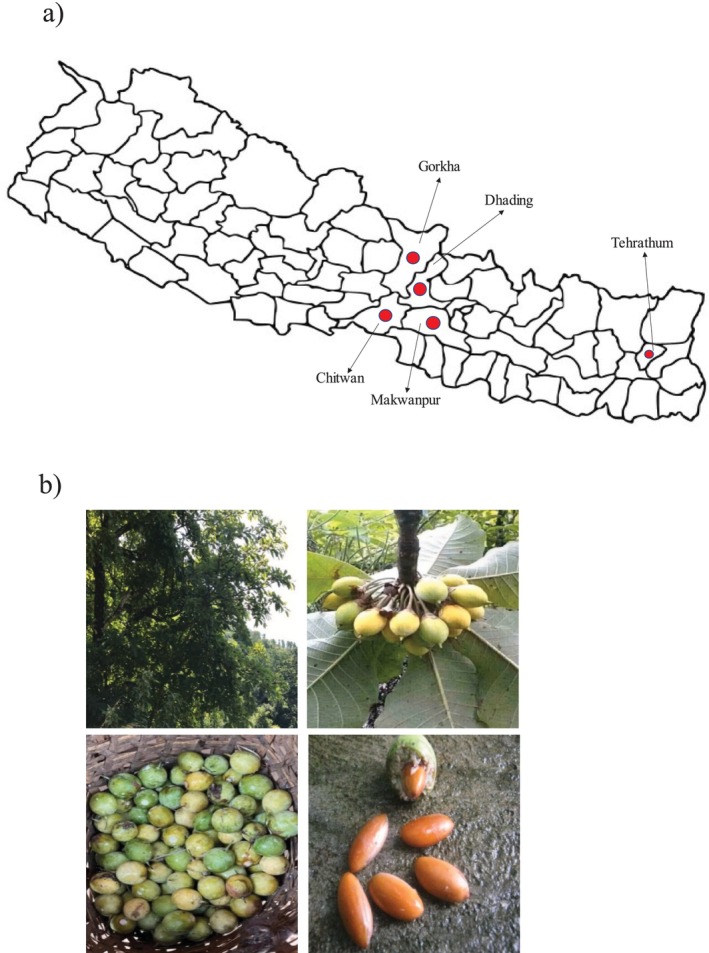
(a) Distribution of chiuri. (b) *D. butyracea* (chiuri) tree; Chiuri fruit; Chiuri fruit and Seed. *Source:* Original photograph.

This tree flourishes on south‐facing slopes in the sub‐Himalayan region, particularly in the Siwalik and higher elevation ranges in Nepal, due to its light requirements. It was discovered to be a part of the natural forest or agricultural vegetation, either alone or in small patches (Bhattarai et al. 2024). As a culturally integrated and multipurpose tree species, it has been seen to flourish in 48 of Nepal's 77 districts (Joshi 2010) for primary healthcare practices, economic generation, and local livelihood (Kunwar and Thakur 2017). There are an estimated 10.8 million chiuri trees in Nepal, spread over most of the country's districts, with Chitwan, Dhading, Tehrathum, Gorkha, and Makwanpur having the highest growth (MEDEP 2014). According to (Joshi 2010), chiuri trees grow naturally on 1900 ha of land in Nepal. The tree density ranges from 37 to 90 trees per ha, with an average of 40 trees per ha (Action 2006). However, local biodiversity, cultural elements, and useful species are all significantly impacted by the current deforestation and degradation, as well as by overexploitation and the invasion of alien invasive plants (MFSC 2014). Bhattarai et al. (2024) observed that in various geographical regions of Nepal, different local varieties of chiuri ( *D. butyracea* ) were cultivated, such as kalo, jire, tomyo, wayo, langhyo, chitiyo, aguwa, pachhuwa, etc. These varieties showed noticeable differences in several traits, including the timing of flowering and fruit ripening, as well as fruit characteristics such as fruit color/seed color, fruit size, leaf color, taste of the juice, and even the onset of defoliation. This variation reflects the rich ethno‐taxonomic diversity shaped by local ecological conditions and traditional cultivation practices. The major districts of distribution of chiuri and its plant parts are shown in Figure 1.

## 3 Harvesting Technique of *D. butyracea*

The *D. butyracea* tree grows on challenging, steep hillsides. Harvesting the chiuri fruit is risky, and climbing the tree is challenging. People frequently fall from trees and suffer severe injuries; occasionally, fatalities happen. The producers use one hand to pick up the fruit and place it in a traditional basket called a doko, which they rest on one shoulder. Typically, women collect the fruit that has fallen to the ground while men scale the trees (Mann 2016).

## 4 Ecosystem Services of Chiuri

A fully grown tree can give 15 kg of seed and 1 quintal of fruit in 20 years. It was found that at an altitude up to 1000 m from sea level, the growing of chiuri tree results in high height and improved yield of fruits and seeds (Tewari et al. 2015). Chiuri trees are also great for soil conservation, as they help bind the soil and can grow well on slopes and poor, barren land, making them ideal for restoring degraded areas (Uprety et al. 2023). In addition, chiuri trees provide food and shelter for bats, birds, bees, and other insects (MEDEP 2010).

A 2015 forest assessment found 18 different uses of chiuri in Nepal's Chure region, with seven related to nutrition. Chiuri also contributes to carbon sequestration, helping in the fight against climate change (Chikanbanjar et al. 2021). Both bees and bats feed on the nectar from chiuri flowers, which are rich in aromatic compounds, playing a key role in promoting co‐evolution and ecological balance. Additionally, the plant has a deep taproot system that supports soil conservation by preventing erosion, aiding in water retention, and contributing to ecosystem stability through gas exchange (Noss 2001).

## 5 Socioeconomic Value of Chiuri in the Nepalese Community

There are significant differences in how the chiuri tree ( *D. butyracea* ) is used by various ethnic groups, professions, and educational levels. Higher educated people and those working in non‐agricultural occupations are less likely to be aware of the applications of chiuri, which is in keeping with global studies that formal education is frequently associated with a loss of traditional ecological knowledge (Bruyere et al. 2016). This change is attributed to educated people moving away from traditional traditions and toward office work (Ouachinou et al. 2019). On the other hand, chiuri is used more diversely and with a deeper understanding among groups with lower levels of formal education, especially those engaged in agriculture and forest‐based livelihoods (Atreya et al. 2018). The Chepang group is notable among these due to their strong ties to the chiuri tree, which they use for culinary, cultural, religious, and medical purposes. Because the tree is so important to their cultural history (Uprety and Asselin 2023), it is traditionally given to girls as a dowry when they get married (Chikanbanjar et al. 2021). The younger generation's declining interest in chiuri‐related customs, however, is causing the Chepang community to become increasingly concerned. In places like Chitwan, where the use of chiuri products has significantly declined, this loss of traditional knowledge and use is apparent (Poudel et al. 2024). In a study conducted by Karki et al. (2023), all respondents reported using at least some portions of the chiuri tree, such as leaves, fruits, and branches, for subsistence reasons, despite the fact that age and gender have been found in several studies to influence the utilization patterns of multipurpose tree species. This emphasizes how prevalent trees are in the everyday lives of communities that have a strong bond with their natural surroundings (Bhattarai et al. 2025).

## 6 Uses of Various Components of the *D. butyracea* Tree

Chiuri products are used commercially in various sectors, including confectionery, pharmaceuticals, vegetable ghee production, candle manufacture, and soap making (Bhattarai et al. 2021). Commercial groups have conducted studies to develop items including chiuri syrup, squash, jam, and cream (Adhikari et al. 2007). Fruits are utilized as nutritional supplements for humans (Shakya 2000). The fruit pulp is consumed by local inhabitants, birds, flies, and wild animals (Bhattarai et al. 2021).

Juicy pulp of ripe fruit is eaten fresh. Juice of the corolla is boiled into a syrupy liquid, which villagers use like syrupy sugar (Manandhar 2002). The fruits and seeds are eaten. The juice extracted from the bark is traditionally used as a medicinal remedy for various ailments, including indigestion, diarrhea, dysentery, asthma, rheumatism, boils, and sinusitis (Bhattarai et al. 2024). The flowers serve for nectar (Dangol et al. 2017). Flowers are an excellent source of honey production (Devkota et al. 2019a; Golay et al. 2021). The honey made by honeybees feasting on chiuri flowers is sold by locals at a reasonable price, providing a good source of income (Bhattarai et al. 2021). The nectar from the blooms is also collected to make jaggery, which is quite valuable (Khanka et al. 2010). A traditional method for manufacturing jaggery from the thick and soft pericarp pulp is to smash the pulp, followed by drying it through boiling. The jaggery is said to be tasty and trades well in the local market (Bhattarai et al. 2021).

The dried powder of the flower and petals is used as a tonic to soothe inflamed throats and to improve lactation. The flowers can also be utilized to produce alcohol (Bhattarai et al. 2021). The ripe fruits are crushed and applied to cure skin diseases in both animals and humans (Devkota et al. 2012). Fresh leaf paste is used to treat oral ulcers and muscle pain (Devkota et al. 2019a; Golay et al. 2021). These leaves have even been traditionally considered beneficial to breastfeeding cattle (Bhattarai et al. 2021). The plant's bark juice is commonly used to treat rheumatism, indigestion, asthma, ulcers, itching, allergies, diabetes, and tonsillitis (Devkota et al. 2012). The roots serve as a tonic. The roots are cleansed, crushed, and soaked in water overnight before being consumed as a tonic (Bhattarai et al. 2021). To treat fever, dried stem bark powder is mixed with water or milk and eaten orally (Bist and Bhatta 2014). Latex is used as an adhesive and is frequently combined with *Ficus roxburghii* (Bhattarai et al. 2024).

The chiuri plant is valued for its diverse applications, particularly the butter extracted from its fruits, which is widely used in household cooking and traditional lamp lighting (Patil et al. 2004). Medicinally, the same butter is reported to alleviate joint‐related ailments (Sherpa 2021). Beyond domestic use, chiuri oil plays a role in small‐scale industries, including soap and candle production (Bhattarai et al. 2021). By‐products such as bark and oil cake have historically been employed in fishing practices due to their toxic properties (Bhattarai et al. 2024). Following processing, the residual cake is repurposed in agriculture as a soil amendment and pest‐resistant fertilizer, and in some cases as livestock feed after detoxification. The residual oil cake remaining after ghee extraction from chiuri seeds is widely applied in agriculture as an organic soil amendment. Farmers, particularly those cultivating banana and paddy crops, value this by‐product for its inherent pest‐deterrent qualities. Owing to its bioactive compounds, the cake functions as a natural control agent against a range of agricultural pests, including insects, rodents, mollusks, and soil‐dwelling nematodes (Chitale et al. 2018).

In Nepal, chiuri trees serve multiple practical purposes, providing fuelwood and sturdy timber, while their foliage is commonly used as livestock feed (Bhattarai et al. 2024). Beyond local utilization, chiuri butter has gained international recognition and is currently supplied to the European cosmetics sector as a raw ingredient meeting COSMOS standard. This butter has been incorporated into a range of personal care products, including creams, ointments, and lotions. Both international and domestic enterprises have engaged in collaborative efforts to develop herbal cosmetic formulations based on chiuri butter, reflecting its growing commercial importance (Bhattarai et al. 2021).

## 7 Physicochemical Composition of *D. butyracea*

In the study by Sakshi Dusat et al. (2017), the moisture content of *D. butyracea* kernels was reported as 22.92%, which was much lower than 58.2% ± 0.642% observed by Mann (2016). Mann (2016) reported protein content (%), fat content (%), ash content (%), carbohydrate content (%) and energy calorific value (Kcal) as 1.24, 0.35, 3.95, 36.25, and 153.11, respectively. Additionally, Sakshi Dusat et al. (2017) found the kernels to contain 46.48% crude fat, 6.81% crude fiber, 2.53% ash, 8.89% crude protein, 23.26% carbohydrate content, and 560 kcal physiological energy. Proximate analysis revealed notable compositional differences between the pulp and seeds of chiuri. The pulp contained lower levels of protein and fat (6.40 and 20.23 g/100 g, respectively) when compared with the seeds, which showed substantially higher values for these components (12.1 and 38.29 g/100 g). In contrast, carbohydrates were more abundant in the pulp (4.05 g/100 g), whereas the seeds exhibited only trace amounts (0.70 g/100 g). Mineral residue, expressed as ash content, was also greater in the pulp (6.45 g/100 g) than in the seeds (2.70 g/100 g) (Dawadi et al. 2023). The nutritional composition of *D. butyracea* and the quantitative analysis of physicochemical composition and minerals in fruit pulp is shown in Tables 2 and 3, respectively.

**TABLE 2:** Nutritional composition of *D. butyracea* .

Constituents	Flower (%)	Constituents	Seeds (%)
Moisture	19.8	Refractive index	1.452–1.462
Protein	6.37	Saponification value	187–197
Fat	0.5	Iodine value	55–70
Total sugar	54.06	Unsaponifiable matter	1–3
Calcium	8.00	Palmitic C 16:0	24.3
Phosphorous	2.00	Stearic acid C 18:0	22.7
Ash	4.36	Oleic acid C 18:0	37.0
		Linolic acid C 18:2	14.3

*Source:* CSIR (2007).

**TABLE 3:** Quantitative analysis of physicochemical composition and minerals in fruit pulp.

Parameters	Reported values	References
Moisture content (%)	40.81	Sherpa et al. (2017)
Acidity (as citric acid) (%)	1.13	Bhattarai et al. (2024)
TSS (°Bx)	18.50	Bhattarai et al. (2024)
Crude protein (%)	3.9–9.55	Bhattarai et al. (2024) and Bhutia et al. (2018)
Crude fat (%)	1.1–1.51	Bhattarai et al. (2024) and Bhutia et al. (2018)
Total ash content (%)	3.37	Sherpa et al. (2017)
Total sugar (%)	7.11–12.21	Bhattarai et al. (2024) and Bhutia et al. (2018)
Reducing sugar (%)	4.53–7.28	Bhattarai et al. (2024) and Bhutia et al. (2018)
Total carbohydrate (mg/g)	21.51	Sherpa et al. (2017)
Non‐reducing sugar (%)	2.98–5.91	Bhattarai et al. (2024) and Bhutia et al. (2018)
Calcium (mg/100 g)	2.89	Singh and Pandey (2021)
Potassium (mg/100 g)	410.42	Singh and Pandey (2021)
Sodium (mg/100 g)	0.68	Singh and Pandey (2021)
Zinc (mg/100 g)	0.08–0.27	Sherpa et al. (2017) and Singh and Pandey (2021)
Copper (mg/100 g)	1.32	Singh and Pandey (2021)
Iron (mg/100 g)	0.40–3.64	Sherpa et al. (2017) and Singh and Pandey (2021)
Magnesium (mg/100 g)	24.93	Singh and Pandey (2021)
Phosphorous (mg/100 g)	52.89	Singh and Pandey (2021)
Manganese (mg/100 g)	0.16	Singh and Pandey (2021)

*Note:* The difference in location can be the reason for the variation of the amount as displayed in the range.

Abbreviation: db, dry basis.

## 8 Processing Method of *D. butyracea* for Oil Extraction

Butter is extracted from chiuri seeds, but the traditional method is labor‐intensive and time‐consuming. Peeling 100 kg of chiuri seeds manually can take an entire day (Devkota et al. 2012). The processing of ghee was once done at the community level, but it is currently done at the household level. To make 1 L of ghee, roughly 18 kg of seeds are needed. Domestic use of processed ghee is being made. Chiuri ghee usage ranged from 2 to 5 kg annually per household in the Chitwan district among the farmers studied. In the traditional process, fruits are first harvested and collected in baskets. The pulp is removed by squeezing the fruits, and the seeds are then sun‐dried for 2–3 days. Once the seeds have been cleaned and allowed to dry, they are ground into a fine powder using a traditional pounder called a “Dhiki.” Over the boiling pan, the powder is steam‐cooked on a perforated plate. A traditional oil expeller known as a “Khole” or “Chepuwa” is then used to extract the oil (Mann 2016), yielding about 25%–30% butter from the seeds. In contrast, modern methods involve drying the seeds in a frying pan and then using an expeller, which increases the butter yield to 40%–45% (Singh et al. 2010). The extracted butter must be refined; unrefined chiuri butter has a creamy, pale‐yellow color, while refined butter appears pure white (Dahal et al. 2021).

## 9 Fatty Acid (FA) Profile and Physicochemical Properties of Chiuri Butter

Chiuri butter is primarily composed of triglycerides (Devkota et al. 2019b). The plant's kernel has a high concentration (> 60%) of oils and fats, as well as high levels of oleic acid (28.00%–31.30%), linoleic acid (4.30%–5.70%), palmitic acid (56.80%–64.10%), and stearic acid (2.40%–3.50%). The yield of oil is 42%–47% of the weight of seeds. It has the consistency of ghee with a white color and a pleasant taste and odor. It has a high titer test. The palmitic acid content (56.6%) is the highest yet observed among seed fats. The oil is a convenient source of natural oleodipalmitin (62%) (Khanka et al. 2009). In the study conducted by (Dahal et al. 2021), palmitic acid, oleic acid, linoleic acid, and stearic acid content were reported as 50%–60%, 30%–40%, 2%–10%, and 0%–5%, respectively. In addition to being utilized for food, oil and fat are also used to make soap and local medications (Bandyopadhyay 2021). The findings of Bandyopadhyay (2021) showed that the oil content of the kernel was determined by the color of the seed coat, stearic acid, and palmitic acid. According to these investigations, there is a great chance that chiuri ghee will find application in the pharmaceutical, confectionery, and cosmetics sectors. The similar composition of oleic acid, linoleic acid, palmitic acid, and stearic acid in chiuri butter was reported by Devkota et al. (2012).

In the physicochemical analysis of chiuri butter, Rautela et al. (2019) reported a moisture content of 0.9%. Several investigations have demonstrated the variation in the acid value of chiuri fat. Sakshi Dusat et al. (2017) reported it as 6.16 mg KOH/g, while Rautela et al. (2019) found it to be 13.01 mg KOH/g. These values were much lower than the 61.86 mg KOH/g reported by Pandey et al. (2021). The iodine value also differed between different studies. Rautela et al. (2019) reported the iodine content of chiuri butter as 23.94 g I_2_/100 g, and Pandey et al. (2021) reported 36.26 g I_2_/100 g, which were both lower than the 99.45 g I_2_/100 g found by Sakshi Dusat et al. (2017). For the peroxide and saponification values, Rautela et al. (2019) reported 5 meq O_2_/kg and 156.24 mg KOH/g. Pandey et al. (2021) observed a peroxide value of 3.14 meq O_2_/kg and a saponification value of 225 mg KOH/g. Sakshi Dusat et al. (2017) had a much higher peroxide value of 39.525 meq O_2_/kg and a saponification value of 176.25 mg KOH/g. Pandey et al. (2021) also reported the ester value as 163.19 mg KOH/g and the liquefaction point as 68°C. According to Sakshi Dusat et al. (2017), the melting point of chiuri fat was 42.23°C, the smoke point was 102.5°C, and the boiling point was 64.66°C.

## 10 Nutritional Properties

Chiuri contains a diverse range of biologically active compounds, notably phenolics along with flavonoids, tannins, terpenoids, glycosidic constituents, and carbohydrates. The presence of these metabolites is associated with notable antioxidant potential and supports the plant's reported anti‐inflammatory and pain‐relieving effects. These pharmacological effects provide scientific support for its traditional medicinal applications (Dahal et al. 2021; Devkota et al. 2019b; Chhetri et al. 2020).

In comparison to seed, pulp was found to have more phytochemicals. Total phenolic content (TPC) in the pulp was 182.26 mg GAEs/100 g, whereas TPC in the seed was 486.08 mg GAEs/100 g mL (Dawadi et al. 2023). Similarly, TPC of aqueous extract of *D. butyracea* was found to be 228.53 mg GAE/g dry extract weight, which may be a significant factor in the diverse biological activities and beneficial for human health promotion (Chhetri et al. 2020). Mann (2016) reported TPC and TFC of chiuri to be 18.96 μg GAE/mg dry material and 16.66 RE μg/mg dry material, respectively. Likewise, the total phenol content and total flavonoid content of aqueous extract of *D. butyracea* bark were found to be 62.16 μg GAE/mg dry extract and 287.6 μg QE/mg dry extract (Chhetry et al. 2022).

Flavonoids are a broad and extremely varied class of naturally occurring phenolic chemicals. The hydroxyl position of flavonoid compounds determines their antioxidant qualities, which are dependent on their ability to donate electrons or hydrogen to a free radical (Pandey et al. 2018). A range of total flavonoid content was found in the *D. butyracea* samples under study, from methanolic bark extract (889.72 μg QE/mg dry extract weight) to hexane pericarp extract (40.63 μg QE/mg dry extract weight). The flavonoid content of the various parts is significantly influenced by the extracting solvent, and even within the same solvent, the content varies from part to part. The flavonoid concentration in the various *D. butyracea* samples used in this investigation is arranged as follows: root bark > pericarp > leaves (Chhetry et al. 2022).

It was observed that the vitamin C concentration of the pulp and seeds was similar (20.70 and 19.08 mg AA/100 g, respectively). Moreover, lycopene, β‐carotene, and trace levels of carotenoids were found in both of these samples (Dawadi et al. 2023). The ascorbic acid content of *D. butyracea* was found to be 22.72 mg/100 g (Pandey et al. 2018). Dawadi et al. (2023) reported 20.70 mg/100 g for pulp and 19.08 mg/100 g for seed. Similarly, Sherpa et al. (2017) found that the ascorbic acid content in chiuri fruit ranged from 16.16 to 33.33 mg/100 g. The difference in location can be the reason for the variation of the amount as displayed in the range.

In a study by Dawadi et al. (2023), lycopene (mg carotenoids/g) of pulp and seed were found to be 0.25 and 0.03, respectively and β‐carotene (mg carotenoids/g) of pulp and seed were found to be 1.06 and 0.29, respectively. Similarly, in a study conducted by Sherpa et al. (2017), chlorophyll content in chiuri fruit ranged from 0.15 to 0.16 mg/100 g, while total carotenoids ranged from 1.41 to 3.00 mg/100 g. The variation in amount might be due to differences in geographical location.

The pulp and seed of *D. butyracea* were found to have respective EC_50_ values of 2207 and 1841.05 μg/mL (Dawadi et al. 2023). Findings of Chhetri et al. (2020) demonstrated that ascorbic acid and aqueous extract of *D. butyracea* bark could lower the DPPH free radical, with respective IC50 values of 2.28 and 8.43 μg/mL. Chhetry et al. (2022) analyzed the antioxidant capacity of different chiuri extracts, which was examined using the DPPH radical scavenging test. Among the samples analyzed, the methanolic extract obtained from the root bark showed the highest antioxidant activity, with an IC_50_ value of 6.1 μg/mL (Chhetry et al. 2022). Similarly, analysis of the fruit extracts revealed that the alcoholic extract was more effective at neutralizing free radicals (IC_50_ = 47.27 μg/mL) than the aqueous extract, which demonstrated comparatively weaker activity (IC_50_ = 69.8 μg/mL) (Pandey et al. 2023). ADBB's ability to neutralize free radicals is likely due to its phenolic compounds. These compounds help protect living systems from oxidative damage by acting as reducing agents, hydrogen donors, and scavenging harmful free radicals and singlet oxygen (Chhetri et al. 2020). The phytochemicals and antioxidants of fresh pulp and the methanolic extract of leaves of *D. butyracea* are shown in Table 4.

**TABLE 4:** Phytochemicals and antioxidants of *D. butyracea* .

Parameters	Per 100 g of fresh pulp weight
Ascorbic acid (mg/100 g)	24.3
Anthocyanin (mg/100 g)	0.6
Total carotenoids (mg/100 g)	7.6
Chlorophyll (mg/100 g)	0.1

Parameters	Methanolic extract of leaves
Total phenolic content (mg GAE/g of dry extract)	665.33
Total flavonoid content (mg QE/g of dry extract)	728
Ascorbic acid (mg/100 g)	48.15
Tyrosinase inhibitory activity (%)	31.07
DPPH IC_50_ (μg/dry extract mL)	6.01

*Source:* Bhattarai et al. (2024) and Giri et al. (2019).

Variations in the varied contents are mostly caused by different extraction methods and different research nations, where variables like location and climate play a big role (Thompson et al. 2024).

## 11 Pharmacological and Functional Bioactivities of *D. butyracea*

Phytoconstituents like alkaloids, tannins, flavonoids, steroids, terpenoids, and palmitic acid are abundant in a variety of plant parts, including the bark, leaf, flower, seed, and fruit. They also contain essential nutrients, including sodium, calcium, potassium, iron, magnesium, zinc, and different types of sugars, all of which contribute to a wide range of pharmacological and therapeutic effects (Anand et al. 2024).

### 11.1 Anti‐Inflammatory Activity

The precise mechanism underlying *D. butyracea* 's anti‐inflammatory properties is unknown. The anti‐inflammatory properties of ADBB, however, might have been aided by the presence of other phytochemical elements, including flavonoids, tannins, terpenoids, and glycosides (Tunon et al. 2009). Because the ADBB had an inhibitory effect on rat hind paw edema in both periods of inflammation, with a particularly pronounced inhibitory effect in the second phase, it may have reduced the inflammatory mediators produced in various stages of inflammation. Paw edema caused by carrageenan was significantly inhibited by the extracts in a dose‐dependent manner. At 200 mg/kg, the ADBB has effects comparable to those of the common medication diclofenac (Chhetri et al. 2020).

### 11.2 Analgesic Activity

ADBB may have a centrally mediated antinociceptive effect, according to the findings of Chhetri et al. (2020). Its analgesic properties could be attributed to the presence of bioactive compounds such as tannins, terpenoids, glycosides, flavonoids, and phenolics. Numerous phytoconstituents have been shown to have analgesic qualities, including triterpenoids, flavonoids, and tannins (Fan et al. 2014). Within the same treatment groups, reaction times increased at all time points as compared to baseline values. In experiments, *D. butyracea* at a dose of 200 mg/kg produced the longest reaction time, comparable to that of the standard drug diclofenac. Additionally, the reaction time increased in a dose‐dependent manner, indicating stronger analgesic effects with higher doses (Chhetri et al. 2020).

### 11.3 Antibacterial and Antifungal Activity of Fruit Extracts

Plant extracts were more efficient against fungi than bacteria. The pulp and seed extracts both showed antifungal efficacy against *Candida albicans* , while itraconazole (10 μg), the positive control, generated a 22 mm zone of inhibition (ZOI). There was no ZOI against any of the species when DMSO was used as the negative control. The highest ZOI was found in *D. butyracea* seed extracts as compared to pulp (Dawadi et al. 2023).

As reported by Tiwari et al. (2020), the antibacterial profile of bark extracts varies significantly by fraction type. While acetone‐based fractions were most effective at inhibiting *Staphylococcus aureus* , the n‐butanol fraction demonstrated the highest efficacy against *Escherichia coli* . Both treatments maintained a ZOI of 15 mm at 1 mg concentrations of extract (Tiwari et al. 2020). Chhetry et al. (2022) studied various parts such as leaf, root bark, and pericarp; and identified the methanolic root bark derivative as the most potent antimicrobial agent, specifically against *S. aureus* (ZOI = 17.33 mm), *S. epidermidis* (ZOI = 14.33 mm), and *K. pneumoniae* (ZOI = 13.0 mm). A notable finding was the universal resistance of *E. coli* across all tested extracts. Bactericidal thresholds varied widely, ranging from a low value of 4.6 mg/mL (aqueous pericarp vs. *S. epidermidis* ) to a much higher 50 mg/mL for the ethyl acetate pericarp against *K. pneumoniae* . Collectively, these results suggest that *D. butyracea* possesses a broad spectrum of bioactive constituents distributed throughout its root bark, leaves, and pericarp (Chhetry et al. 2022).

Similarly, in a study conducted by Maharjan et al. (2024), no fungal growth was observed at 10%, 15%, and 20% concentrations of *D. butyracea* seed extracts against *Fusarium oxysporum* f. sp. *cubense* R4 (FocR4) and *Rhizoctonia* spp. Due to the harmful impacts of synthetic fungicides on both farmers' health and agriculture, the use of botanical and plant‐derived fungicides for controlling fungal diseases has increased. In support of sustainable agricultural practices, *D. butyracea* seeds may contain potent antifungal compounds that could be utilized for managing fungal infections in agricultural contexts (Maharjan et al. 2024).

### 11.4 Nitric Oxide Radical Scavenging Assay

The aqueous extract of *D. butyracea* bark showed nitric oxide scavenging activity, although it was slightly lower than that of the standard compound curcumin, with IC50 values of 148.33 and 102.99 μg/mL, respectively (Chhetri et al. 2020). This free radical scavenging effect is likely due to its antioxidant properties, as the extract can act as a hydrogen donor, reducing agent, and scavenger of singlet oxygen and free radicals (Kim et al. 1999). By effectively interacting with oxygen, the extract helps reduce the formation of nitrate and nitrite ions (Chhetri et al. 2020).

## 12 Commercial Value of *D. butyracea*

**FIGURE 2:**
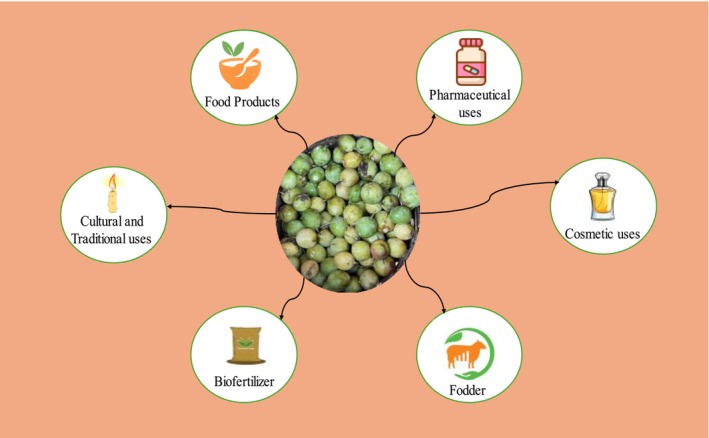
Diverse applications of chiuri.

There is significant potential and plenty of resource base (Figure 2) to establish and expand chiuri‐based micro‐ and small enterprises. Nepal has the potential to produce around 37,245 t of chiuri butter and 17,285 t of honey from its existing chiuri trees. The value of chiuri butter alone is estimated at over NPR 5 billion (about USD 38 million as of March 2024) (MEDEP 2010). It is estimated that Nepal exports 20 tons of chiuri butter to Europe annually (Dahal et al. 2021). Promising opportunities include the production of chiuri butter products, beekeeping and honey processing, and extraction of nectar and pulp, as well as the creation of eco‐friendly paper plates and handcrafted furniture (Oli et al. 2024). Both the fruits and seeds are edible once detoxified and can serve as food for humans or feed for animals. The yellow‐colored fruits are a good source of carbohydrates and can also be used in industrial processes for alcohol purification (Swain et al. 2007).

All the fruits of the tree ripen at the same time, and there is no established system to extend their shelf life (Dahal et al. 2021). According to previous studies (Chhetri et al. 2022; Parajuli et al. 2022; Dangal et al. 2023; Dangal et al. 2021), fruits with high water content can be used to prepare fruit‐based products. While seed fats can be utilized for lighting lamps, cooking, and to make hair oil, candles, and soap, fruits are used as dietary supplements (Devkota et al. 2012). The nectar from the flowers is used to produce sugar candy (Joshi 2010).

The growing global demand for natural, sustainable, and plant‐based ingredients positions *D. butyracea* as a strong candidate for commercialization within circular bioeconomy models. Functional foods can also be developed from by‐products like seed cake and oil residues of *D. butyracea* , which are rich in bioactive compounds. Innovative product development could include energy bars, powders, or oils. Further possible uses include the development of nutraceuticals, fortified foods like snacks, and specialized beverages that strengthen immunity and provide numerous health benefits.

Future commercialization strategies should include standardization of extraction and refining processes, safety and toxicological validation for food and pharmaceutical applications, regulatory approval as a novel or traditional plant‐based ingredient. Overall, incorporating *D. butyracea* into value chains beyond its traditional use has significant prospects for economic growth, sustainable resource utilization, and product innovation. Various applications of *D. butyracea* are shown in Figure 2.

## 13 Ethnomedical Uses of *D. butyracea*

Different ethnic groups in Nepal use different sections of the chiuri plant as food and medicine. Ghee made from chiuri seeds, which are high in fats and oils, is referred to as Vanaspati ghee (Meena 2016). Chiuri ghee is utilized in antipyretic, anti‐inflammatory, analgesic, headache, and rheumatism treatments (Mishra 2015). According to Watanabe et al. (2013), seed fat, also known as chiuri butter, is used as an emollient for wintertime chapped hands and feet and to cure burns, boils, pimples, rheumatism, and headaches.

The best benefits for bacterial dysentery have been found with leaf extract. Leaf extracts also treat bronchitis and Cushing's illness (Yadav et al. 2012). In order to cure eczema and pain, leaves are used as cataplasm (Bisht et al. 2018). Women and children gather the fallen flowers and let them dry in the sun. Most commonly, flowers are used as cough remedies, tonics, and to promote lactation (Mishra 2015). In cases of head disorders (pitta diseases), flowers are also used as nasal drops. Additionally, flowers are used as a stimulant, a refrigerant, and to treat gastritis, frequent urination, and to protect the liver (CSIR 2007).

It has also been discovered that stems and barks have therapeutic value as an antidote for diabetes, rheumatism, tonsillitis, itching, and snakes (Prashanth et al. 2010). The bark of this tree has been traditionally used to treat a wide range of ailments, including asthma, indigestion, ulcers, itching, bleeding, limb contractions, wounds, rheumatic pain, tonsil inflammation, leprosy, and diabetes (Rijal 2011). Additionally, according to Meena (2016), a mixture of bark powder with ghee and honey is believed to enhance sexual vigor and overall vitality. The bark extract and blossoms of Chiuri have anti‐diabetic properties (Mishra 2015).

Diverse biological activities such as antioxidant effects, antifungal effects, feeding deterrents, and insect growth inhibitory effects have been reported from the fruit extract (Adhikari‐Devkota et al. 2023). The bark of *D. butyracea* contains several bioactive compounds, including triterpenoids like α‐amyrin acetate, β‐amyrin acetate, and friedelin; steroids such as α‐spinasterol; steroid glycosides like the β‐D‐glucoside of β‐sitosterol; and 3β‐palmitoxyolea‐12‐en‐28‐ol (Chhetri et al. 2020). These phytochemicals are known for a range of beneficial biological activities, including anticancer, antioxidant, anti‐inflammatory, analgesic, and wound‐healing effects, which have attracted significant interest from the pharmaceutical industry (Choi et al. 2012).

## 14 Toxicity of *D. butyracea*

Chhetri et al. (2020) evaluated the safety of the aqueous bark extract of *D. butyracea* at doses of 50, 100, and 200 mg/kg body weight. The extract was proved to be safe across all doses, as no toxic effects or deaths were recorded.

## 15 Utilization Pattern of *D. butyracea* in Different Areas in Nepal

Bhattarai et al. (2025) collected a total of 2023 use reports, covering 21 different emic uses of 10 parts/products of the chiuri tree ( *D. butyracea* ). These uses were grouped into six broader categories for easier understanding. Among all plant parts, fruits, leaves, and stems were mentioned in every report (100% citation), showing their importance. Butter (57.03%) and honey (50.54%) were also frequently cited. Bark was mainly valued for its medicinal uses, while stems and branches were commonly used as fuelwood and timber. Leaves were fed to livestock during food shortages and also used in religious rituals. Flowers were collected mainly for honey production. Fruits were eaten as a seasonal treat and sometimes used to make wine. Seeds were especially important for extracting butter and producing oil cake, which was further used as organic fertilizer and pesticide. Although less commonly mentioned (7.71%), whole chiuri trees were also used for cultural purposes.

Overall, chiuri was used across six major categories, i.e., medicinal, religious, cultural, subsistence, industrial, and culinary, showing its multipurpose value. Most use reports (1440) fell under the subsistence category, followed by medicinal (240) and religious (213) uses. Subsistence uses included 12 different local applications that helped support rural livelihoods. Common examples include eating the fruit, using leaves as animal fodder, and burning branches for firewood; this was reported in every location, with a 100% citation score. Other subsistence uses included bark latex, flower‐based products like syrup, honey, and jaggery, fruit‐based drinks, and oil cake for fertilizer and pest control (Bhattarai et al. 2025).

The use of chiuri varied depending on geography and ethnic background. In Makawanpur (Central Nepal), all 10 parts/products were reported to be used, followed by nine in nearby Chitwan. The Chepang community used all parts of the tree—fruit, leaf, stem, butter, honey, oil cake, bark, latex, nectar, and even the whole plant—across all six categories. In contrast, only three parts (fruit, leaf, and stem) were reported in the Panchthar district (Eastern Nepal), particularly among the Limbu ethnic group (Bhattarai et al. 2025).

## 16 Conclusions

There is growing evidence that the antioxidant activity of *D. butyracea* provides therapeutic effects when consumed. Despite its great potential for a variety of uses, such as food, medicine, cosmetics, and more, this versatile plant is underexplored and not widely promoted in accordance with its worth. All components of this plant are rich in nutrients and possess bioactive properties, including anti‐inflammatory, antifungal, antioxidant, and antibacterial properties.

Regardless of its usefulness, its use is confined to a regional scope, which to some extent makes it more difficult to set a price, determine its worth, and market it. In addition, the plant is neglected by the government in terms of research and support, whereby it can boost the economy of the area and the welfare of the citizens of the region. With respect to the plant, little effort has been made to study it in order to open up opportunities, discover its potential, and fit it into a larger picture.

Future studies can focus on the development of innovative functional food utilizing the by‐products of chiuri by developing a safe food‐grade extraction process and evaluating possible effects on food systems. Future prospects could also include performing higher‐level research, raising awareness at the locality about the importance of this plant, and enhancing production processes in such a way that they will aim at the extraction of its more popular therapeutic, cultural, or economic value.

## Funding

The authors have nothing to report.

## Ethics Statement

The authors have nothing to report.

## Conflicts of Interest

The authors declare no conflicts of interest.

## Data Availability

The authors have nothing to report.
